# Functional Nanomaterials and Polymer Nanocomposites: Current Uses and Potential Applications

**DOI:** 10.3390/ijms232112713

**Published:** 2022-10-22

**Authors:** Raghvendra Singh Yadav

**Affiliations:** Centre of Polymer Systems, Tomas Bata University in Zlin, Trida Tomase Bati 5678, 76001 Zlin, Czech Republic; yadav@utb.cz; Tel.: +420-576-031-725

In the present Special Issue “Functional Nanomaterials and Polymer Nanocomposites: Current Uses and Potential Applications”, two review articles and nine original research articles are published. The published review article by M.S.A. Darwish et al. [1] presents the research advances on polymeric nanocomposites for environmental and industrial applications. Further, C.V. Rocha et al. [2] provide a review article on current advances in the development and biomedical applications of PLGA-based materials.

In a published research article in this Special Issue, M. Aviv et al. [3] describe the behavior of the double-fluorinated Fmoc-Phe derivatives, Fmoc-3,4F-Phe and Fmoc-3,5F-Phe, and the influence that the position of single fluorine has on the self-assembly process and physical characteristics that the material produces. Moreover, S. Stojanov et al. [4] reported the incorporation of vaginal lactobacilli into electrospun nanofibers to achieve a prospective solid vaginal delivery system, and further, the fluorescent proteins were incorporated to differentiate them and allow their tracking in the future probiotic-delivery investigations. C. Miyamaru et al. [5] developed CaCO_3_-coated vesicles by biomineralization and further their utilization as carriers of drug-delivery systems. In this Special Issue, M.B. Stie et al. [6] described mucoadhesive electrospun nanofiber-based hybrid system with the controlled and unidirectional release of desmopressin. Anju et al. [7] investigated highly efficient electromagnetic interference shielding of Cu_x_Co_1−_xFe_2_O_4_ (x = 0.33, 0.67, 1) magnetic nanoparticle-based polyurethane nanocomposites with reduced graphene oxide. Further, C.-Y. Wu et al. [8] report conductive supramolecular polymer nanocomposites with tunable characteristics to manipulate cell growth and functions. J. Fèvre et al. [9] describe, in this Special Issue, chelating polymers for targeted decontamination of actinides and application of PEI-MP to Hydroxyapatite-Th(IV). Furthermore, Z. Wang et al. [10] describe mono-sized anion-exchange magnetic microspheres for protein adsorption. In addition, M. Park et al. [11] investigated the impact of hexagonal boron nitride insulating layers on the driving ability of ionic electroactive polymer actuators for lightweight artificial muscles.
